# Effect on Neonatal Mortality of Newborn Infection Management at Health Posts When Referral Is Not Possible: A Cluster-Randomized Trial in Rural Ethiopia

**DOI:** 10.9745/GHSP-D-16-00312

**Published:** 2017-06-27

**Authors:** Tedbabe Degefie Hailegebriel, Brian Mulligan, Simon Cousens, Bereket Mathewos, Steve Wall, Abeba Bekele, Jeanne Russell, Deborah Sitrin, Biruk Tensou, Joy Lawn, Joseph de Graft Johnson, Hailemariam Legesse, Sirak Hailu, Assaye Nigussie, Bogale Worku, Abdullah Baqui

**Affiliations:** a United Nations Children's Fund (UNICEF), New York, USA.; b John Snow Research and Training Institute, Inc., Yangon, Myanmar.; c London School of Hygiene and Tropical Medicine, London, UK.; d Save the Children International, Addis Ababa, Ethiopia.; e Save the Children, Washington, DC, USA.; f UNICEF, Addis Ababa, Ethiopia.; g World Health Organization, Windhoek, Namibia.; h Bill & Melinda Gates Foundation, Seattle, WA, USA.; i Ethiopian Pediatrics Society, Addis Ababa, Ethiopia.; j International Center for Maternal and Newborn Health, John Hopkins Bloomberg School of Public Health, Baltimore, MD, USA.

## Abstract

**Background:**

The World Health Organization recently provided guidelines for outpatient treatment of possible severe bacterial infections (PSBI) in young infants, when referral to hospital is not feasible. This study evaluated newborn infection treatment at the most peripheral level of the health system in rural Ethiopia.

**Methods:**

We performed a cluster-randomized trial in 22 geographical clusters (11 allocated to intervention, 11 to control). In both arms, volunteers and government-employed Health Extension Workers (HEWs) conducted home visits to pregnant and newly delivered mothers; assessed newborns; and counseled caregivers on prevention of newborn illness, danger signs, and care seeking. Volunteers referred sick newborns to health posts for further assessment; HEWs referred newborns with PSBI signs to health centers. In the intervention arm only, between July 2011 and June 2013, HEWs treated newborns with PSBI with intramuscular gentamicin and oral amoxicillin for 7 days at health posts when referral to health centers was not possible or acceptable to caregivers. Intervention communities were informed of treatment availability at health posts to encourage care seeking. Masking was not feasible. The primary outcome was all-cause mortality of newborns 2–27 days after birth, measured by household survey data. Baseline data were collected between June 2008 and May 2009; endline data, between February 2013 and June 2013. We sought to detect a 33% mortality reduction. Analysis was by intention to treat. (ClinicalTrials.gov registry: NCT00743691).

**Results:**

Of 1,011 sick newborns presenting at intervention health posts, 576 (57%) were identified by HEWs as having at least 1 PSBI sign; 90% refused referral and were treated at the health post, with at least 79% completing the antibiotic regimen. Estimated treatment coverage at health posts was in the region of 50%. Post–day 1 neonatal mortality declined more in the intervention arm (17.9 deaths per 1,000 live births at baseline vs. 9.4 per 1,000 at endline) than the comparison arm (14.4 per 1,000 vs. 11.2 per 1,000, respectively). After adjusting for baseline mortality and region, the estimated post–day 1 mortality risk ratio was 0.83, but the result was not statistically significant (95% confidence interval, 0.55 to 1.24; *P*=.33).

**Interpretation:**

When referral to higher levels of care is not possible, HEWs can deliver outpatient antibiotic treatment of newborns with PSBI, but estimated treatment coverage in a rural Ethiopian setting was only around 50%. While our data suggest a mortality reduction consistent with that which might be expected at this level of coverage, they do not provide conclusive results.

## Abstract (precis)

Health Extension Workers (HEWs), in general, properly provided antibiotic treatment of possible severe bacterial infections in newborns at the health post level. But only about half of newborns estimated to have infections in the intervention area received treatment by HEWs, and home visits and referrals declined in the final months of the study. Cluster-level analysis suggests a mortality reduction consistent with this level of treatment coverage, although the finding did not reach statistical significance.

## INTRODUCTION

Serious infections such as sepsis, meningitis, and pneumonia are estimated to cause more than 550,000 newborn deaths each year.^1^ Most of these deaths could be averted by preventive measures, such as hygienic practices and cord care, as well as by timely identification of signs of infection and treatment with appropriate antibiotics.^2^^,^^3^ In low-income, high-mortality settings, however, access to hospitals where treatment can be provided is often difficult or impossible for many newborns with signs of infection.^4^^–^^7^

Based on new evidence from the African Neonatal Sepsis Trial (AFRINEST) and the Simplified Antibiotic Therapy Trial (SATT),^8^^–^^10^ the World Health Organization (WHO) recently provided guidelines for the treatment of possible severe bacterial infections (PSBIs) in infants where referral to hospital is not feasible.^11^ These guidelines recommend that trained health care providers give outpatient treatment for newborns and young infants 0–59 days of age with PSBI using simplified regimens; for infants with clinical severe infection, the recommended regimen is injectable gentamicin plus oral amoxicillin, and for infants with isolated rapid breathing, oral amoxicillin only. These recommendations were classified as “strong” according to WHO Grading of Recommendations Assessment, Development, and Evaluation (GRADE) criteria.^12^ The primary outcome in the carefully conducted AFRINEST and SATT trials was “treatment failure,” including clinical outcomes in addition to death. However, there is limited experience implementing this approach in routine, primary health care settings, and the mortality impact is not known.

WHO recently provided guidelines for the treatment of possible severe bacterial infections in infants where referral to hospital is not feasible.

In Ethiopia, facility-based care, and in particular hospital-based care, is not accessible to much of the population. Only 40% of women receive antenatal care, only 15% deliver with a skilled attendant, and just 12% receive postnatal care within 2 days after birth.^13^ In 2003, Ethiopia launched the national Health Extension Program to address access issues.^14^ Under this program, nearly 34,000 Health Extension Workers (HEWs) have been deployed to 14,000 health posts, the most peripheral level of the health system.^15^ The Health Extension Program includes 16 packages of mostly preventive health interventions delivered by HEWs at health posts and through community outreach. The program initially provided limited curative services, but in mid-2010 it introduced integrated community case management (iCCM) for treatment of pneumonia, diarrhea, malaria, and severe acute malnutrition in children 2–59 months.^16^

Between 2008 and 2013, the Community-Based Interventions for Newborns in Ethiopia (COMBINE) trial evaluated the impact of a regimen of intramuscular gentamicin and oral amoxicillin—a regimen similar to the new WHO recommendations—given by HEWs in Ethiopia to newborns and young infants with signs of PSBI when referral was not possible. The purpose of this article is to present findings on the feasibility and mortality impact of this approach. When this trial was conceived, families were required to seek care for infections in newborns and young infants at higher-level facilities. With the high neonatal mortality rate in Ethiopia (37 deaths per 1,000 live births according to the 2011 Demographic and Health Survey^17^), treating newborns closer to home could avert many preventable deaths.

The COMBINE trial evaluated the impact of a simple antibiotic regimen given by HEWs in Ethiopia to newborns and young infants with signs of possible severe bacterial infection when referral was not possible.

## METHODS

### Study Setting

COMBINE was conducted in a population of 640,000 in 3 zones—East Shoa and West Arsi in Oromia Region and Sidama in Southern Nations, Nationalities, and People's Region (SNNPR)—that are demographically and socioeconomically similar to Ethiopia's agrarian regions where 85% of the country's people live. Ethiopia's tiered primary health care system includes hospitals (approximately 1 per 100,000 population), health centers (about 1 per 25,000), and health posts (about 1 per 5,000). Health centers are usually staffed by several nurses. The satellite health posts are staffed by 2 HEWs, who are females with 10th-grade education recruited from the communities they serve. The Health Extension Program provides HEWs with 1 year of training.

### Study Design

COMBINE was a 2-arm, cluster-randomized trial evaluating the impact of making newborn infection management available at health posts when referral to health centers was not possible.

Clusters comprised a health center with 5 or 6 health posts and their catchment population; each cluster had around 1,000 births annually. We assumed the neonatal mortality rate was 32 deaths per 1,000 live births at the start of implementation of the intervention in 2010 (representing a 22% reduction in rural neonatal mortality from the 2005 DHS,^18^ which we expected after activities to strengthen the Health Extension Program had been completed in all study areas), with 50% of deaths occurring within 24 hours after birth.^19^ Few deaths within 24 hours after birth would be due to infection and those that were would be difficult to identify and treat. Assuming a coefficient of variation of 0.24 and postulating 33% mortality reduction, we sought to detect a reduction in post–day 1 neonatal deaths from 16.0 to 10.7 per 1,000 day 1 survivors. Eleven clusters were required per arm for 80% power.^20^

#### Randomization and Masking

Clusters were randomized 1:1 stratified on region and using restriction to ensure arms were balanced in population size, annual number of births, baseline neonatal mortality rate, and proportion of HEWs resident in their village.^21^ Allocation was not masked, although survey teams were blinded to minimize interviewer bias.

#### Intervention Description

Before the start of the study, community meetings were held to orient religious and administrative leaders and other community representatives on the study's purpose and to obtain community consent to conduct the study in these areas. Because HEWs already have many responsibilities, we decided to introduce volunteers to help the HEWs make the desired number of home visits we wished to achieve in our study. In both arms, a community-led process was held to select female volunteers (1 per 100–150 population) from groups of women active in health promotion activities.

**FIGURE 1:**
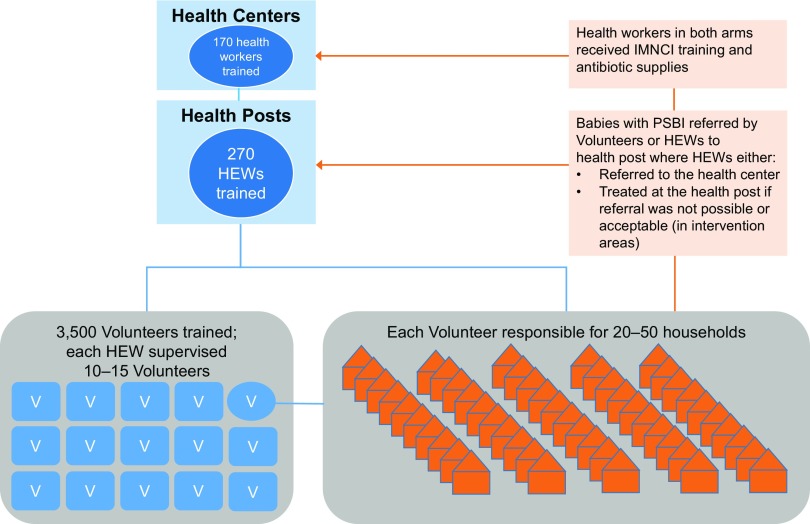
Relationship Between Health Extension Workers, Volunteers, and Households in Identifying and Managing Newborn Infections, Rural Ethiopia Abbreviations: HEW, Health Extension Worker; IMNCI, integrated management of neonatal and childhood illnesses; PSBI, possible severe bacterial infection; V, Volunteer.

We trained a total of 3,500 female volunteers and 270 HEWs. Volunteers received 4 days of training on what to do during home visits, including counseling families about the importance of antenatal care, danger signs for women and newborns that should prompt care seeking, birth preparedness, clean delivery, and healthy newborn care practices that prevent infection and other illnesses. Training also included how to identify and refer sick newborns to health posts. Volunteers were not paid but received transportation and lunch allowances during trainings. HEWs received 4 days of training on home visits (same content as the volunteer training), 3 days on volunteer support, and 6 days on iCCM, including assessing and referring infants under 2 months with PSBI signs to health centers and case management for sick children 2 months and older. The iCCM training was included because national rollout had not yet been completed. The HEWs in the intervention areas also received 1 additional day of training on the study treatment algorithm (described below), administration of medicine to newborns, and injection safety. In accordance with Ethiopia's Health Sector Development Plan, each HEW supervised and supported 10–15 volunteers, with each volunteer responsible for visiting 20–50 households (Figure 1).^14^

Both volunteers and HEWs conducted home visits to counsel families about danger signs and care seeking and to identify infants with possible severe bacterial infection.

For both arms, the schedule of home visits consisted of 2 visits during pregnancy from volunteers, after identifying the pregnancy through monthly surveillance or receiving notification from the community or family, plus 1 pregnancy visit by HEWs, after receiving notification from volunteers. Postnatal visits were scheduled with volunteers on the day of birth, day 3, and day 7, and with HEWs within 2 days of birth and on day 4. During counseling, volunteers and HEWs used pictorial materials adapted from the national Family Health Card.

In both arms, HEWs referred newborns with PSBI to health centers. At the health center, in line with the integrated management of neonatal and childhood illnesses (IMNCI) standard of care, health workers administered pre-referral antibiotics to the newborns with PSBI and then referred the newborns to the hospital. To ensure high-quality referral care, health centers were supplied with job aids and antibiotics, and health center staff were trained on IMNCI using the national 7-day curriculum.

**FIGURE 2:**
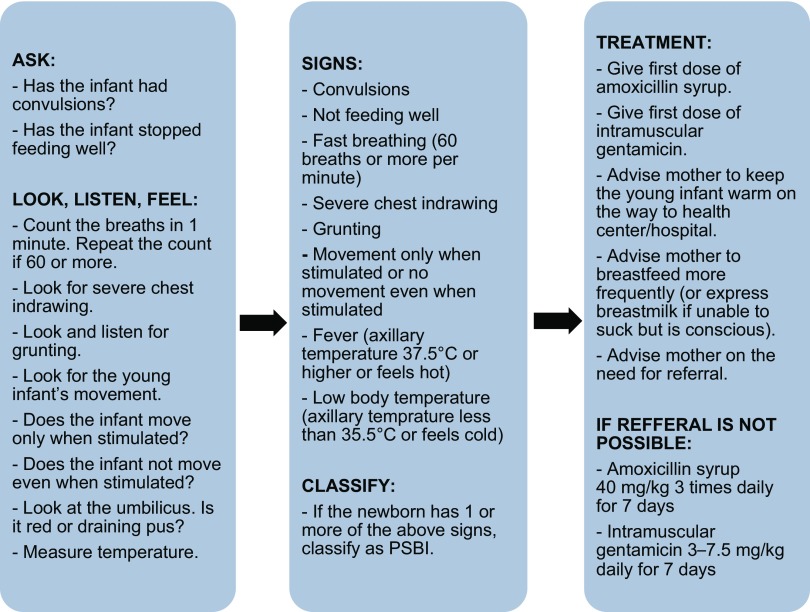
Algorithm for Assessment, Classification, and Treatment of Newborn Infection by Health Extension Workers at Intervention Health Posts Abbreviation: PSBI, possible severe bacterial infection.

In the intervention arm only, HEWs were trained to treat PSBI in newborns and young infants at health posts, if referral was not possible or acceptable. This study used a similar clinical algorithm for PSBI to the simple algorithm identified in the WHO Young Infants Clinical Signs Study (YICSS) as predicting severe illness in infants aged 0–2 months, which consisted of 7 signs: history of difficulty feeding, history of convulsions, movement only when stimulated (or no movement even when stimulated), respiratory rate of 60 breaths or more per minute, severe chest indrawing, temperature of 37.5°C or more, temperature below 35.5°C.^23^ The COMBINE study added grunting as an eighth danger sign (Figure 2). HEWs treated babies diagnosed with PSBI with daily gentamicin injections for 7 days and oral amoxicillin (administered by caretakers) 3 times daily for 7 days. Intervention health posts were supplied with job aids and antibiotics for treating neonatal PSBI. Additional community meetings were held in intervention clusters to raise awareness of the availability of treatment for sick newborns at health posts to encourage care seeking. Table 1 summarizes the study inputs for each of the 2 arms.

**TABLE 1.** Description of Newborn Infection Management Intervention by Arm and Health System Level, Rural Ethiopia, 2008–2013

	Control	Intervention
**Community and Household**		
Half-day meetings to orient religious and administrative leaders and other community representatives on the study purpose and to obtain community consent to conduct study in these areas	Yes	Yes
1-day meetings with religious and administrative leaders and other community representatives to select female volunteers for the study	Yes	Yes
4-day training for HEWs and volunteers on conducting home visits to counsel women and families on the importance of antenatal care, facility delivery, and postnatal care; birth preparedness (saving money, planning place of delivery, transport, and blood donor); healthy newborn care practices; recognition of danger signs in mothers and newborns that require prompt care seeking; postpartum family planning; and identification and referral of sick newborns	Yes	Yes
2-day refresher training for volunteers to reinforce initial 4-day training	Yes	Yes
3-day training for HEWs on how to work with and support volunteers	Yes	Yes
Identification of pregnant women by study volunteers	Yes	Yes
Pregnancy and postnatal home visits by HEWs and volunteers per training	Yes	Yes
1-day meetings to raise community awareness of the availability of treatment for sick newborns at health posts	No	Yes
**Health Post**		
6-day training for HEWs on assessment and referral of newborns with signs of PSBI and case management for sick children older than 2 months (using national iCCM materials)	Yes	Yes
1-day training for HEWs on treatment of newborns with signs of PSBI when referral is not possible or acceptable	No	Yes
Monthly meetings among PO, HEWs, and volunteers to reinforce counseling skills, referral of sick newborns, and reporting on home visits	Yes	Yes
Supervision of HEWs by POs	Yes	Yes
Provision of antibiotics and supplies for PSBI case management to health posts	No	Yes
Treatment of newborns with signs of PSBI by HEWs, if referral to the health center was not possible or acceptable	No	Yes
Monthly PSBI case management review meetings between POs and HEWs	No	Yes
Documentation of symptoms, diagnosis, treatment initiation, and referrals for sick young infants by HEWs on iCCM registers	Yes	Yes
Tracking of the number and timing of antibiotic doses and outcome by HEWs	No	Yes
**Health Center**		
7-day training for health center staff on IMNCI using national curriculum	Yes	Yes
Provision of antibiotics and supplies for PSBI case management to health centers	Yes	Yes
Documentation of symptoms, diagnosis, treatment initiation, and referrals for sick young infants by health center staff on IMNCI registers	Yes	Yes

Abbreviations: HEW, health extension worker; iCCM, integrated community case management; IMNCI, integrated management of neonatal and childhood illnesses; PSBI, possible severe bacterial infection; PO, project officer.

The study used a simple clinical algorithm with 8 danger signs to identify infants with possible severe bacterial infections.

Project Officers (POs) with nursing backgrounds were employed by the study in both arms, each supporting 2–4 health posts through twice-monthly visits for monitoring and supervision. POs, HEWs, and volunteers also met monthly to review home visit coverage, documentation, and counseling and assessment skills. In intervention areas, additional monthly meetings were held for PSBI data review and clinical mentoring.

Health systems strengthening activities, including the initial trainings of HEWs, volunteers, and health center staff and provision of antibiotics and supplies to health posts and health centers, were completed in both arms by March 2010. Also in March 2010, HEWs and volunteers started conducting home visits to counsel and support caregivers on exclusive breastfeeding, keeping the baby warm, clean cord care, and handwashing, as well as to identify and promptly refer cases of PSBI. Clusters were randomized in August 2010, and treatment of PSBI by HEWs if referral was not possible or acceptable was implemented in the intervention arm from July 2011 to the end of the study (June 2013). The time lag between randomization and the start of the intervention was due to unforeseen external delays unrelated to the restriction or sampling criteria.

#### Primary Outcomes

The primary study outcome was post–day 1 neonatal mortality (deaths on days 2–27 after birth). In the absence of ongoing demographic surveillance, data were collected via surveys of all consenting households in the study area. Baseline data were collected from June 2008 to May 2009. A 6-year truncated pregnancy history was collected from consenting women ages 15–49. Information on socioeconomic status, knowledge, practices, and care seeking was collected from women who had delivered during the previous 60 days. Verbal consent was obtained. The endline survey (conducted between February 2013 and June 2013) used the same methodology except it employed a 3-year pregnancy history to reduce the data collection workload. We estimated mortality based on births and deaths in the year preceding each survey to ensure endline estimates covered a period when the intervention was in place and fully functional.

In the absence of ongoing demographic surveillance data, we collected neonatal mortality data through household surveys.

#### Data Collection and Analysis

Baseline survey data were collected by staff hired by the project who were supervised by the POs. The POs conducted the verbal autopsies. For the endline survey, we hired an independent company to collect the data. The baseline and endline household surveys employed several levels of data quality assurance. Periodic observation of interviews, re-interviews, review meetings, questionnaire review, and hand tallies of selected indicators were done in the field. Before data entry, forms were checked for completeness, legibility, linkage, and consistency. Data were double-entered with range and consistency checks. We used 4 survey modules to collect the necessary data: (1) a household listing administered to the head of the household; (2) a pregnancy history administered to women of reproductive age identified in the household listing; (3) a questionnaire on newborn care practices administered to women who had delivered within the past 60 days; and (4) verbal autopsy administered to caregivers who reported a newborn death.

We also collected routine monitoring data. Volunteers and HEWs submitted forms for every identified pregnancy/birth and home visit. The validity of this system was assessed through a household survey in November 2011 using multistage cluster sampling and interviewing women with a birth within the previous 3–4 months.

POs extracted data on symptoms, diagnosis, treatment initiation, referrals, and outcomes from iCCM registers at health posts and IMNCI registers at health centers. We introduced a separate form at intervention health posts to track the number and timing of antibiotic doses, which were not recorded in the iCCM register. From mid-2011 through early 2012, POs accompanied HEWs on home visits when possible and independently assessed neonates to evaluate the quality of HEWs' assessments. POs stopped parallel assessments after sufficient data were available to demonstrate HEWs were skilled in conducting assessments.

Data were analyzed using Stata version 12 (www.stata.com). Baseline descriptive demographic information was analyzed to examine the comparability of the arms. The primary analysis was by intention-to-treat using cluster-level summary data due to the small number of clusters. From the pregnancy history, babies born 1 year preceding baseline or endline survey and those surviving day 1 were identified, and cluster-level mortality risks for days 0–27, days 0–1, and days 2–27 were computed. (We grouped newborns that died less than 1 day after birth and on the day after birth to ensure we captured all deaths within 24 hours.) Analyses compared each of the 3 different mortality risks between intervention and comparison arms at endline, adjusted for baseline risks. Analyses of covariance were performed in which the dependent variable was the log of the cluster-specific mortality risk at endline. Covariates in the model were treatment arm, region, and the log of cluster-specific baseline mortality risk. Results of fitting these models provided an estimate of mortality risk ratios in the intervention arm compared with the control arm adjusted for baseline mortality, 95% confidence intervals, and a test of the null hypothesis of no difference between arms. A secondary analysis, based on individual-level data using generalized estimating equations to account for intra-cluster correlation, was also performed.

To estimate coverage of pregnancy and postnatal home visits, routine data were used to identify the number of women and newborns receiving pregnancy and postnatal home visits in 2011. Endline survey data were used to estimate denominators (number of births in 2011 for pregnancy visits, number of live births in 2011 for postnatal visits). Including all women who received a visit during pregnancy in 2011 in the numerator would overestimate coverage of pregnancy visits since some women visited in 2011 gave birth in 2012. Practically, we could not exclude women who gave birth after 2011 since we did not have the date of birth for all women. Therefore, we assumed the number of women visited in 2011 and gave birth in 2012 was roughly similar to the number of women who received a first pregnancy visit in 2010 and gave birth in 2011. Thus, we excluded women who received a first pregnancy visit in 2010 from the numerator for coverage of pregnancy visits. Since the window for scheduled postnatal visits in our study was narrow (within 7 days of birth), we assumed only a very small number of women received a postnatal visit in 2011 but gave birth in 2010, and therefore did not exclude any women from our numerator for coverage of postnatal visits. Data from the 2011 validation survey were analyzed for comparison to estimates obtained from the routine data, accounting for clustering using Taylor's linearization method.^22^

Data from the iCCM register were extracted to obtain the number of newborns presenting to health posts and health centers, the number of cases of PSBI, symptoms, referrals, and outcomes. Because a separate form was introduced at intervention health posts to track the number and timing of antibiotic doses and outcome, unique identifying information was used to link this information to data extracted from the iCCM register. Data extracted from the iCCM register were also used to calculate the case fatality rate and estimate the number needed to treat to prevent 1 death. We compared POs' and HEWs' assessment of signs of PSBI using cross-tabulations.

#### Ethical Approval and Role of Funding Source

The Ethiopian Science and Technology Agency and the London School of Hygiene & Tropical Medicine Ethics Committee approved the study protocol. The study was registered at ClinicalTrials.gov, number NCT00743691.

The funder had no role in study design, data collection, data analysis and interpretation, writing of the report, or decision to submit for publication. The corresponding author had full access to all the data in the study and had final responsibility for the decision to submit for publication.

## RESULTS

### Background Characteristics

Most baseline characteristics were similar between arms (Table 2). The general fertility rate was slightly higher in the intervention arm than the control arm (169 live births per 1,000 women of reproductive age vs. 163 per 1,000, respectively), and a lower proportion of women reported having any education (26% in the intervention arm compared with 33% in the control arm).

**TABLE 2.** Basic Sociodemographic Characteristics at Baseline (2008–2009), Rural Ethiopia

	Comparison	Intervention
**No. of households interviewed**	**58,944**	**60,408**
No. of residents per household, median	5	5
**No. of women of reproductive age interviewed**	**58,497**	**56,733**
General fertility rate (no. of live births per 1,000 women of reproductive age)	163	169
**No. of women with a live birth in past 1 year**	**9,531**	**9,600**
Age in years of women with live birth in past 1 year, mean (SD) (range)	28 (6) (14–50)	28 (6) (14–50)
No. of lifetime live births prior to interview among women with live birth in past 1 year, median	4	4
Neonatal mortality rate among women with live birth in past 1 year (deaths during days 0–27 per 1,000 live births)	33.6	35.0
**No. of women with a live birth in past 60 days**	**1,371**	**1,358**
Percentage of women with live birth in past 60 days who ever attended school	33%	26%
No. of years of education among women with live birth in past 60 days who ever attended school	4	4
Wealth quintiles among women with live birth in past 60 days, No. (%)		
Lowest	287 (21%)	253 (19%)
2nd	258 (19%)	280 (21%)
3rd	274 (20%)	273 (20%)
4th	261 (19%)	280 (21%)
Highest	280 (20%)	262 (19%)
Missing	11 (1%)	10 (1%)

Abbreviation: SD, standard deviation.

### Home Visit Coverage

Routine data collected from volunteers and HEWs suggested high home visit coverage in both arms. In 2011, the first full year of home visit implementation, an estimated 84% of women in comparison areas were reported to have received pregnancy visits and 77% postnatal visits, compared with 92% and 77% of women, respectively, in intervention areas. The volunteers conducted most of the home visits in both arms (82% of pregnancy visits and 88% of postnatal visits). The validation survey, conducted in November 2011, produced similar home visit coverage estimates as the routine monitoring data in the comparison arm (84% for pregnancy visits and 74% for postnatal visits), but somewhat lower coverage estimates in the intervention arm (76% for pregnancy visits and 71% for postnatal visits). We were not able to link survey data and routine data from the same mothers to determine which source was more valid for capturing home visits. There was a decline in home visits toward the study's end, evident in the routine monitoring data and also reflected in endline survey data among women with births in the preceding 60 days. In comparison areas, 38% of these women reported pregnancy visits and 27% reported postnatal visits; in intervention areas, 43% reported pregnancy visits while 36% reported postnatal visits.

Volunteers conducted most of the home visits in both arms of the study.

### PSBI Cases

**FIGURE 3:**
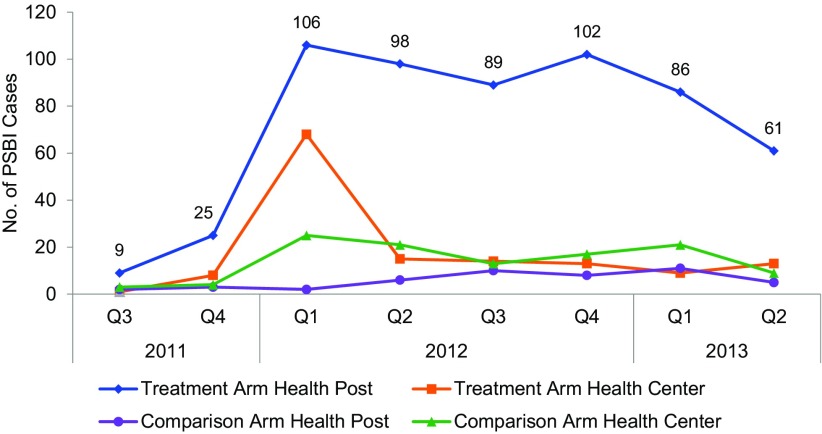
Number of Newborns With at Least 1 Sign of PSBI Presenting at Health Centers or Health Posts by Study Arm, Rural Ethiopia, July 2011–June 2013 Abbreviation: PSBI, possible severe bacterial infection.

The number of PSBI cases presenting to health posts increased dramatically in intervention areas, from only 9 cases in the third quarter of 2011 to a high of 106 cases in first quarter of 2012 (Figure 3). During the remainder of 2012, the number of PSBI cases presenting to health posts remained relatively steady at about 90 to 100 cases each quarter, but the numbers appeared to decline in 2013 with a low of 61 cases. In contrast, in comparison areas there was little change in the number of PSBI cases presenting to health posts with no more than 13 cases seen per quarter. In both arms, there was an initial spike in PSBI cases seen at health centers during the first quarter of 2012, but thereafter few cases were seen at health centers in either arm.

The number of cases with possible severe bacterial infections presenting to health posts increased dramatically in intervention areas, but a decline was evident toward the end of the study period.

Of 1,011 sick newborns presenting at intervention health posts from July 2011 to June 2013, 576 (57%) were identified by HEWs as having 1 or more signs of PSBI. About half (53%) of the PSBI cases were referred to the health post by a volunteer, while the rest were either self-referred (33%), referred by an HEW after identification during a home visit (11%), or had missing data (4%). Of the 576 identified cases, 521 (90%) were treated by HEWs at health posts, 22 (4%) received the first treatment dose by HEWs and were then referred to health centers, 19 (3%) were referred by HEWs to health centers without treatment at the health post, and 14 (2%) had missing data.

57% of sick newborns presenting at intervention health posts were identified by HEWs as having 1 or more signs of possible severe bacterial infection.

90% of cases identified by HEWs as having possible severe bacterial infection were treated by HEWs at the health posts.

### HEW Performance

Table 3 compares identification of 7 danger signs (all but rapid breathing) by HEWs and POs. HEWs generally performed well, identifying all danger signs identified by POs except in 5 cases (2 cases of fever, 2 cases with low temperature, and 1 case with grunting). Compared with POs, HEWs may have slightly overdiagnosed PSBI, identifying 10 babies with PSBI (13 danger signs) whom POs did not identify. Ability to identify rapid breathing was assessed by comparing the number of breaths per minute recorded by HEWs and POs for 855 babies. HEWs recorded the same breathing rate plus or minus 2 breaths for 591 babies (69%) (data not shown). HEWs counted 3 or more breaths per minute over the PO's count for 112 babies (13%), and HEWs counted 3 or more breaths fewer than POs for 152 babies (18%). Few of these assessments were in babies with a sign of PSBI.

**TABLE 3.** Comparison of Assessments of Newborn Danger Signs by HEWs and POs, Rural Ethiopia, 2011–2012

Danger Signs	Identified by Both the HEW and PO	Identified by HEW Only	Identified by PO Only	Agree Sign Not Present
Convulsions	2	0	0	825
Not feeding well	4	1	0	811
Chest in-drawing	14	4	0	840
Grunting	8	5	1	832
Lack of movement	42	2	0	810
Fever	10	0	2	797
Low temperature	13	1	2	769

Abbreviations: HEW, Health Extension Worker; PO, Project Officer.

### PSBI Signs

Of 521 PSBI cases treated at intervention health posts by HEWs, 163 (31%) had fast breathing as the only symptom. In this trial, children with isolated fast breathing received the same treatment as children with other signs of PSBI. Twenty-five treated cases (5%) had a history or presence of convulsions, a sign of critical illness, and 237 (45%) of treated cases had more than 1 sign of PSBI. Only 6 cases (1%) had grunting, which is not part of the YICSS algorithm, as the only danger sign.

### Treatment Completion Among PSBI Cases

Of the 521 cases treated by HEWs at the health post, 414 (79%) could be linked using unique identifying information to data on administered doses, which was collected separately. Among the linked cases, 408 (99%) received 7 gentamicin doses per study protocol. If we assume no other PSBI cases completed treatment, the treatment completion rate among all PSBI cases treated at the health posts would be 79%. However, an additional 79 babies were recorded as receiving 7 gentamicin doses but could not be linked with the administrative data. Therefore, the completion rate is likely greater than 79%. Among all 521 treated cases, 10 (2%) died. All 10 babies who died had multiple signs of infection.

At least 79% of cases with possible severe bacterial infection treated by HEWs at the health post completed the treatment regimen.

### Mortality Impact

**FIGURE 4:**
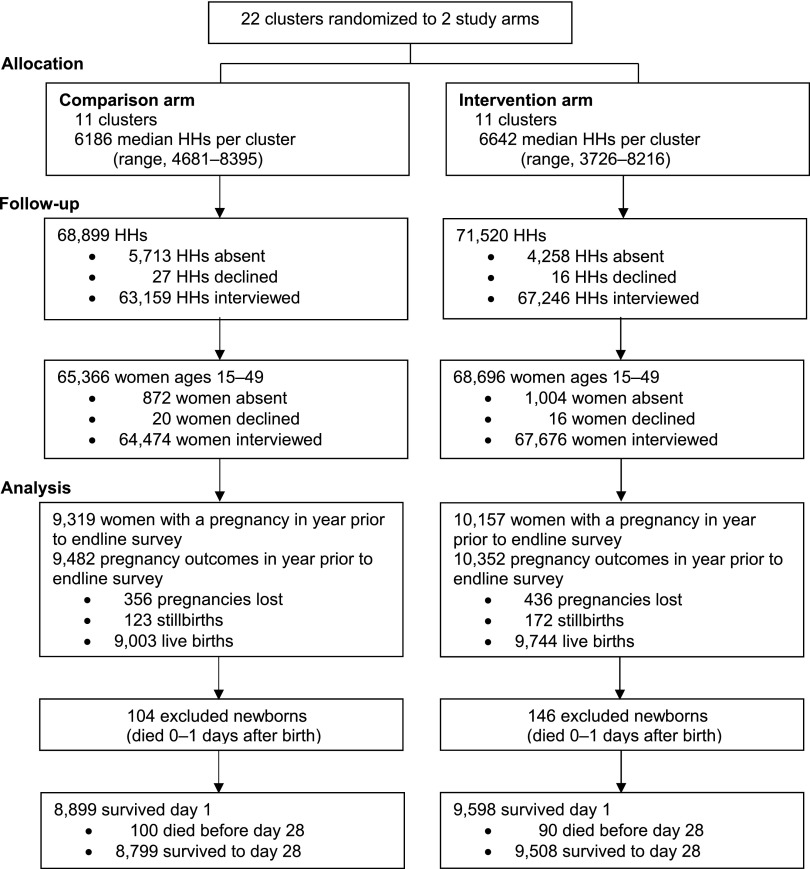
Study Allocation and Participants Interviewed and Included in Main Outcome Analysis Using Endline Survey Abbreviation: HH, household.

Mortality impact analyses were based on the pregnancy history data from the household surveys. In comparison areas, 98.6% of identified women of reproductive age were interviewed for the endline survey; 9,319 women reported a pregnancy in the preceding year, resulting in 9,003 live births. In intervention areas, 98.5% of identified women of reproductive age were interviewed; 10,157 women reported a pregnancy in the preceding year, resulting in 9,744 live births (Figure 4). The total number of births across both arms (18,747) was slightly lower than the expected number used to calculate sample size (1,000 per cluster or 22,000 total).

Deaths on days 0–1 declined in both arms, with no evidence the treatment intervention was associated with a reduction in these very early neonatal deaths (adjusted risk ratio [RR], 1.04; 95% confidence interval [CI], 0.70 to 1.55; *P*=.83) (Table 4). Post–day 1 mortality declined more in the intervention arm than in the control arm. After accounting for baseline mortality risk and region, results from the cluster-level analysis are consistent with a 17% reduction in post–day 1 mortality, but the results were not statistically significant (RR, 0.83; 95% CI, 0.55 to 1.24; *P*=.33) (Table 4). Results from the secondary individual-level analysis suggest a greater reduction (27%) in post–day 1 all-cause mortality in intervention areas, but these results were also not statistically significant (RR, 0.72; 95% CI, 0.49 to 1.06; *P*=.09).

**TABLE 4.** Neonatal Deaths at Baseline (2008–2009) and Endline (2013) by Study Arm and Timing of Deaths, Rural Ethiopia

Timing of Neonatal Deaths	Comparison		Intervention		Intervention vs. Comparison	
	Denominator N	Mortality Rate (n)	Denominator N	Mortality Rate (n)	Adjusted Risk Ratio^a^ (95% CI)	*P* Value
Days 0–27						
Baseline	9,531 live births	33.6 (320)	9,600 live births	35.0 (336)	0.94 (0.72, 1.22)	.61
Endline	9,003 live births	22.7 (204)	9,744 live births	24.2 (236)		
Days 0–1						
Baseline	9,531 live births	19.4 (185)	9,600 live births	17.4 (167)	1.04 (0.70, 1.55)	.83
Endline	9,003 live births	11.6 (104)	9,744 live births	15.0 (146)		
Days 2–27						
Baseline	9,346 surviving day 1	14.4 (135)	9,433 surviving day 1	17.9 (169)	0.83 (0.55, 1.24)	.33
Endline	8,899 surviving day 1	11.2 (100)	9,598 surviving day 1	9.4 (90)		

Abbreviation: CI, confidence interval.

a Endline intervention vs. comparison, adjusted for baseline mortality risk and region.

Post–day 1 mortality declined more in the intervention arm than in the control arm.

If we assume mortality was reduced 22%, which is midway between the estimated mortality reduction from the cluster-level analysis (17%) and the individual-level analysis (27%), then we estimate the intervention averted about 20 deaths in the intervention arm in the year prior to the endline survey. In this 1-year period (January 2012 through December 2012), 359 PSBI cases were treated by HEWs at health posts, suggesting that the number needed to treat to prevent 1 death is around 18. Given 9,744 live births in the intervention arm 1 year prior to the endline survey, we estimate that about 3.7% of live births received antibiotic treatment by HEWs at health posts.

## DISCUSSION

To our knowledge, this trial is the first to evaluate the implementation and mortality impact of outpatient newborn infection treatment using a simplified regimen at the most peripheral level of a health system in a low-resource country. Bang et al. tested a package of home visits with home-based newborn infection management by project-employed community health workers and reported a 62% mortality reduction.^4^ Baqui et al. used project-employed workers to provide home visits with home-based treatment of newborn infections when referral was not possible and reported a 34% mortality reduction.^5^ Both these studies were conducted in places with a much higher baseline neonatal mortality rate (62 deaths per 1,000 live births and 47 deaths per 1,000 live births, respectively) compared with the baseline rate in our study (34.3 deaths per 1,000 live births in intervention and comparison areas combined), and neither was implemented through the existing health system. A 2003 meta-analysis of trials of community-based pneumonia management estimated a 27% mortality reduction and 42% pneumonia-specific mortality reduction among neonates; only 5 studies provided data on neonatal mortality and most of the data points came from Bang et al.^24^ A study in Zambia of management of common perinatal conditions by traditional birth attendants concluded that offering a first dose of antibiotics at home without strengthening referral care was insufficient to reduce mortality from infection.^25^

Our study suggests HEWs in Ethiopia are able to correctly identify signs of PSBI, and there is high treatment compliance among PSBI cases treated by HEWs. While our data are consistent with a reduction in post–day 1 neonatal mortality following the introduction of newborn infection management by HEWs at health posts, we must acknowledge the inconclusive nature of the statistical evidence. A recent meta-analysis of 22 studies estimated a global PSBI incidence risk of 7.6%, with a slightly higher incidence (8.2%) among studies using the YICSS algorithm and a slightly lower incidence (6.2%) in studies in sub-Saharan Africa (6 study sites in Africa).^26^ In our study, which used a case definition of PSBI that was very close to the YICSS algorithm, an estimated 3.7% of live births were treated by HEWs at health posts, suggesting that somewhere in the region of half of all babies with PSBI were treated at health posts. Thus, despite substantial inputs, it appears that the intervention tested in our study did not remove all barriers to accessing care. Furthermore, home visit coverage and the number of PBSI cases presenting to the health post appeared to decline in the final months of study, indicating that the study inputs may not have been sufficient to sustain a change in care-seeking behavior as project inputs were reduced. Our point estimate suggests a 17% reduction in post–day 1 neonatal mortality in the context of around 50% treatment coverage. Extrapolating this further suggests that complete treatment coverage could have had the potential to reduce post–day 1 deaths by around one-third. This figure appears plausible given that an estimated 27% of all neonatal deaths in Ethiopia are due to severe infections^1^ and that the proportion of post–day 1 deaths due to infections will be considerably higher. In the context of declining neonatal mortality and improved preventive measures, it will become increasingly difficult to detect the contribution of infection treatment to mortality reduction. To achieve a detectable mortality impact in such a scenario, sustained high treatment coverage would be necessary.

About half of all babies with possible severe bacterial infection were treated by HEWs at health posts, suggesting the need for additional interventions to remove barriers to accessing care.

In most settings where implementation of outpatient infection management would be appropriate, systems strengthening inputs are needed, including in-service training, supervision, commodities, monitoring, and community mobilization. In our study, we engaged, trained, and supervised volunteers to support HEWs in improving care seeking among community members and in identifying and referring PSBI cases. In fact, these volunteers conducted most of the home visits and referred about half of the PSBI cases seen at intervention health posts. HEWs, who have multiple responsibilities and must spend time at health posts, did not make as many home visits, and few cases seen at health posts had been referred by HEWs during a home visit. These findings demonstrate that successful case detection is highly dependent on having a cadre with both the skills to identify and refer cases and the time to conduct multiple home visits. The importance of the volunteers in our study was evident as the number of identified cases declined toward the end of the study because the volunteers reduced their activity levels in anticipation that newborn treatment would not continue after the end of the study. In addition, introduction of the government's new volunteer strategy called the Health Development Army created confusion and sidelined many existing volunteers. Given that about a third of the PSBI cases in our study were self-referred, in the longer run, community education and mobilization efforts, aside from home visits, could change care-seeking norms and thus diminish the need for active case detection. But until norms change, effective, active case detection is required to achieve meaningful mortality reductions. In our study, we estimated around 18 PSBI cases needed to be treated at the health post level to prevent 1 death.

Successful case detection of infants with infection requires a cadre with both the skills to identify and refer cases and the time to conduct home visits.

The case fatality rate among cases treated by HEWs in this study was 2%, which is in line with the case fatality rate for babies treated by community health workers in Baqui et al.'s community-based trial (1.4%)^27^ and the case fatality rates for cases of clinical severe illness treated in all arms of the SATT (2%) and AFRINEST (1.5%) trials.^9^^,^^10^ Approximately 31% of cases in our study had isolated fast breathing, which were excluded from the definition of clinical severe illness in SATT and AFRINEST, and we would expect low mortality in this group even without treatment. On the other hand, 5% of treated cases had convulsions. Such cases were classified as having critical illness and were excluded from SATT and AFRINEST. A further 45% of cases in our study had multiple signs of illness compared with 38% in SATT and 13% in AFRINEST. Despite some differences in case mix compared with these other studies, the case fatality we observed in our routine program setting seems to be broadly in line with what has been observed in carefully controlled trials.

Previous studies focused on home-based treatment because it was believed that many families would not be willing to take their newborns outside the home for treatment, especially in a country such as Ethiopia where there is a traditional practice of keeping newborns at home.^28^ Initially, our study had low care seeking at health posts (Figure 3). In response, we conducted qualitative interviews to identify barriers to care seeking, which elicited such barriers as fear of the “evil eye,” beliefs that exposure to the sun made newborns sick, and a tradition of keeping the baby away from strangers (including health workers) until the newborn was blessed by spiritual leaders. The project then implemented community mobilization activities, which likely drove the observed improvements in home visits and care seeking in the intervention arm.

Distance also often presents an insurmountable barrier to accessing care in settings where service provision is sparse, transport infrastructure weak, and populations poor.^29^^,^^30^ However, our study suggests families are willing to seek care outside the home over several days—specifically, for 7 days of treatment in this study—when services are close. In both arms of the study, counseling families on danger sign recognition and care seeking was strengthened with treatment for PSBI offered at the health center level (in addition to the health post level in the intervention arm if referral was not possible or acceptable), but few families sought care for their sick newborns at these higher-level facilities. While there was an initial spike in cases seen at intervention health centers, mirroring the increase in cases seen at intervention health posts, this trend was not sustained. The inability to sustain the increase at health centers could be explained by the policy of giving a first antibiotic dose at health centers and then referring to the higher-level hospitals, which requires time and resources from families. In contrast, there was a large increase in care seeking for newborn illness at health posts in intervention areas, where newborns with PSBI could receive the full course of antibiotic treatment if referral was not possible or acceptable, with high treatment completion. Therefore, these results from the COMBINE study demonstrate the importance of improving access to newborn health care by making treatment available closer to home.

Families may be willing to seek care for their infants outside the home over several days when services are nearby.

### Strengths and Limitations

Study strengths include the cluster-randomized design and large geographic area covered. Further, the study was designed to be pragmatic with case management delivered through the existing system, although some additional inputs were provided. Study limitations include incomplete monitoring records on treatment adherence; we had linked information on the number of doses administered for only 79% of cases at intervention health posts, and it proved impossible to collect this information at health centers. In addition, mortality impact data were dependent on survey recall data and mothers were often unable to recall the precise timing of birth and death. Therefore, we excluded deaths on day 0 (day of birth) and day 1, which allowed us to exclude all deaths within 24 hours, but we may have included some deaths that occurred after 24 hours. Furthermore, the relatively small number of clusters, 11 per arm, resulted in a very wide confidence interval around our primary outcome effect estimate. Finally, although baseline neonatal mortality rate, the criteria used for randomization, was similar between arms (4% higher in intervention areas at 35.0 per 1,000 live births compared with 33.6 per 1,000), the baseline post–day 1 mortality rate was 24% higher in intervention versus comparison areas (17.9 per 1,000 live births compared with 14.4 per 1,000, respectively).

## CONCLUSIONS

The Ethiopian Ministry of Health, with partner support, has started to scale up the COMBINE model of community-based newborn care with newborn infection management by HEWs at health posts.^31^ However, the new cadre of volunteers called the Health Development Army may not do as much active case detection as the volunteers did in the COMBINE study. Based on the experience of COMBINE, we expect low coverage of treatment until care-seeking norms change. In other settings where most families do not seek care for sick newborns, the introduction of treatment needs to be accompanied by a comprehensive plan to change care-seeking behavior. Adaptation of the COMBINE model in other countries must also consider the available health delivery platforms. Ethiopia already had an established cadre of community-based health professionals with 1 year of basic training—HEWs. These HEWs cover a sufficiently large catchment population to see enough cases to justify resources for training, supervision, and monitoring on neonatal infection management and to maintain their skills through continued practice. In other contexts, a similar community-based cadre may not be available and thus it may be more appropriate to introduce outpatient treatment of neonatal infections at primary health facilities.

## Notes

**Peer Reviewed**
